# The conserved and high *K-to-Na* ratio in sunflower pollen: Possible implications for bee health and plant-bee interactions

**DOI:** 10.3389/fpls.2022.1042348

**Published:** 2022-11-01

**Authors:** Michał Filipiak, Morgan W. Shields, Sarah M. Cairns, Megan N. C. Grainger, Stephen D. Wratten

**Affiliations:** ^1^ Institute of Environmental Sciences, Faculty of Biology, Jagiellonian University, Kraków, Poland; ^2^ Bio-Protection Research Centre, Lincoln University, Lincoln, New Zealand; ^3^ School of Science, University of Waikato, Hamilton, New Zealand

**Keywords:** ecological stoichiometry, ionomics, elementome, pollen, pollination, pollinivory, potassium, sodium

## Abstract

Sodium (Na) concentrations are low in plant tissues, and its metabolic function in plants is minor; however, Na is a key nutrient for plant consumers. Previous studies have thus far focused on Na concentration. Nevertheless, a balanced potassium (K) to Na ratio (*K:Na*) is more important than Na concentration alone since food with high *K:Na* has detrimental effects on consumers irrespective of Na concentration. Therefore, plants may actively regulate *K:Na* in their tissues and products, shaping plant-insect interactions. Studies considering nutritional aspects of plant-insect interactions have focused on nonreproductive tissues and nectar. In this study, we consider pollen as serving a primary reproductive function for plants as well as a food of pollinivores. Plants might regulate *K:Na* in pollen to affect their interactions with pollinivorous pollinators. To investigate whether such a mechanism exists, we manipulated Na concentrations in soil and measured the proportion of K, Na, and 13 other nutrient elements in the pollen of two sunflower (*Helianthus annuus*) cultivars. This approach allowed us to account for the overall nutritional quality of pollen by investigating the proportions of many elements that could correlate with the concentrations of K and Na. Of the elements studied, only the concentrations of Na and K were highly correlated. Pollen *K:Na* was high in both cultivars irrespective of Na fertilization, and it remained high regardless of pollen Na concentration. Interestingly, pollen *K:Na* did not decrease as pollen increased the Na concentration. We hypothesize that high *K:Na* in pollen might benefit plant fertilization and embryonic development; therefore, a tradeoff might occur between producing low *K:Na* pollen as a reward for pollinators and high *K:Na* pollen to optimize the plant fertilization process. This is the first study to provide data on pollen *K:Na* regulation by plants. Our findings broaden the understanding of plant-bee interactions and provide a foundation for a better understanding of the role of the soil-plant-pollen-pollinator pathway in nutrient cycling in ecosystems. Specifically, unexplored costs and tradeoffs related to balancing the *K:Na* by plants and pollinivores might play a role in past and current shaping of pollination ecology.

## Introduction

Plant tissues and products differ in their chemical profiles but have one thing in common: high concentrations of K and low concentrations of Na (Kaspari, 2020). This unique plant feature poses a challenge to consumers of plant matter, and an unbalanced *K:Na* in food reduces the fitness of plant consumers (Kaspari, 2020; Kaspari, 2021). The *K:Na* ratio in bee food resources may be more important than the overall Na concentration since Na and K are physiologically bound (Kaspari, 2020). Animals need a balanced *K:Na* to maintain their phosphate homeostasis and sensing; they also require Na-K ATPases, osmoregulatory ability, the ability to adapt to cold and heat and to maintain their microbiomes, which also need a balanced *Na:K* (Pedersen and Zachariassen, 2002; Dow, 2017; Kaspari, 2020). Overall, an unbalanced *K:N*a in food reduces the fitness of plant consumers.

Regarding pollinivores, acute bee paralysis and even death may be caused by an excessively high *K:Na* ratio in Western honey bee food [(Horn, 1985); see also Kaspari (2020) who provided the context and biological explanation for this finding]. Furthermore, the ratio of *K:Na* in pollen consumed by wild bee larvae may affect bee fitness, as feeding experiments have shown that concentrations of both K and Na in wild bee larval food influence fitness-related traits, such as mortality, body mass and cocoon development (Filipiak and Filipiak, 2020; Filipiak et al., 2022). These effects may potentially influence wild bee populations and communities (Filipiak et al., 2022). Petanidou (2007) suggested that pollinivores may be attracted to food sources having balanced *K:Na*. Indeed, in contrast to earlier studies focusing on concentrations of individual elements (Lau and Nieh, 2016; Bonoan et al., 2017; Bonoan et al., 2018; de Sousa et al., 2022), our recent study (Cairns et al., 2021) showed that the Western honey bee preference for “dirty water”, i.e., water enriched with nutrients, is driven by the *K*:*Na* ratio rather than by the Na concentration alone.

Correspondingly, it was suggested that plants may regulate concentrations of Na in their tissues and products in response to their interactions with herbivores and pollinators (Kaspari, 2020; Kaspari, 2021; Finkelstein et al., 2022). A literature review of the elemental composition of pollen suggested that K concentrations are among the most stable of the 11 elements for which data are available, while Na concentrations are the most variable (Filipiak et al., 2017). Additionally, plant tissues and products with higher concentrations of Na, are preferred by herbivores (e.g. (Nicolson and W.-Worswick, 1990; Borer et al., 2019); see (Finkelstein et al., 2022) for experimental confirmation). We therefore hypothesize that plants regulate *K:Na* in pollen by increasing or decreasing the concentration of Na in pollen. Such regulation might serve either (1) to encourage pollen consumption, making pollen a nutritionally balanced reward for pollinivores and improving animal pollination services, or (2) to discourage pollinivores from pollen eating, making pollen nutritionally inadequate for consumers and relying on wind pollination or specialized pollination services by physiologically adjusted consumers.

In this brief research report, we explored the idea that pollinivore-pollinated plant actively regulates *K:Na* in pollen while maintaining proportions of other elements for the overall nutritional balance of pollen for pollinators. To that end we increased an Na concentration in the soil assuming that such an increase would result in an increased Na concentration in pollen produced by a pollinivore-pollinated plant. We hypothesized that an increase in the Na concentration in pollen would result in a decreased *K:Na* ratio whilst maintaining the overall proportion of the remaining elements (i.e., elements other than K and Na), therefore improving the entire nutritional quality of pollen for pollinivores. To test this hypothesis, we investigated whether and how nutritional quality changes with an increased Na concentration in sunflower (*Helianthus annuus* L.) pollen with a focus on the *K:Na* ratio. Nutritional quality was reflected in the pollen stoichiometric phenotype, i.e., the proportion of vital nutrient elements [for detailed explanation see (Filipiak and Filipiak, 2022)]. Sunflower was chosen because it is a common monoculture and mass-flowering crop that provides large amounts of foraging resources for bees (Todd et al., 2016). We discuss the results within the context of a theory of sodium ecology, as introduced by Kaspari (2020), with a special focus on the nutritional needs of pollinivorous pollinators, i.e., bees.

## Materials and methods

### Experimental setup

To evaluate pollen nutritional quality, we measured the concentrations and proportions of 15 vital elements in pollen: Na, K, P, Mg, Ca, Fe, Mn, Cu, Zn, B, V, Cr, Co, Ni and Sr (note that studied heavy metals are among vital elements (Fraústo da Silva and Williams, 2001; Williams and Rickaby, 2012; compare with Jager et al., 2013; Świątek et al., 2020; Filipiak and Bednarska, 2021). This multi-element approach allowed us to study the correlation of Na and K concentrations in pollen with concentrations of other elements contributing to the overall nutritional quality of pollen. To consider as much natural variation in the concentrations of nutrients as possible, we used two sunflower cultivars grown under different conditions.

Plants were grown in a closed greenhouse in May-October 2020 (winter-spring) in Lincoln, New Zealand, and involved two sunflower (*Helianthus annuus* L.) cultivars, yellow pygmy (hereafter YP) and Baltic, with three Na treatments comprising NaCl (high: 10.728 g/pot, low: 7.152 g/pot, and control: no added Na). There were six treatments in total, each with 10 replicates. The NaCl concentrations were calculated based on findings from Bhatt and Indirakutty (1973) on Na uptake by sunflowers. Therefore, the concentrations of NaCl added were based on how much Na sunflowers had been found to mobilize from soil. We used two staggered sowing dates (SDs, SD 1: 20/05/2020, and SD 2: 10/06/2020) to minimize the risks of environmental disturbance and inadequate pollen sample size. NaCl was administered as crystals sprinkled over the soil, followed by a 5-second, moderate-intensity flow of tap water to allow the NaCl to enter the soil profile. NaCl was applied at growth period stage 3 and reproductive stage 4 (R4) (Schneiter and Miller, 1981) at the beginning of pollen development when the developing pollen grains acquired nutrients during microsporogenesis. Growth period stage 3 (Robinson, 1971) was defined as the point at which the inflorescence head was visible, and yellow petals were visible underneath the unfurling bracts of the inflorescence. Reproductive stage R4 (Schneiter and Miller, 1981) was characterized as when the inflorescence began to open up and, when viewed from above, small ray florets were visible on the inflorescence head. Each replicate consisted of one 14 L pot with two plants per pot (in case one plant died or did not flower). Pots were randomized and watered 2-3 times per week.

Additionally, we grew plants under the same conditions and treatments as above but with 12 replicates and three sowing dates (SD 1: 20/05/2020, SD 2: 10/06/2020, and SD 3: 01/07/2020). Replicates at growth period stage 3 (Robinson, 1971) and reproductive stage R4 (Schneiter and Miller, 1981) were moved from the greenhouse to a fenced area with frost cloth to reduce wind and frost damage. Replicates were arranged in a randomized block design of 12 blocks, with one replicate of each treatment per block. Blocks were 1 m apart, and replicates within each block were 0.5 m apart.

### Pollen collection and elemental analysis

Inflorescences from the greenhouse were harvested in August at reproductive stage 5.5 (R5.5) when half the disc inflorescences were undergoing anthesis and pollen was available (Schneiter and Miller, 1981). Inflorescences from the field were harvested in September-October at R5.5 after one week of field exposure. Under laboratory conditions, inflorescences were tapped to allow pollen to drop onto greaseproof baking paper in a shallow tray. The pollen was then carefully transferred from the paper into sterile sieves to remove contaminants and stored in Falcon tubes. If less than 20 mg of pollen was collected from a single replicate, then it was pooled with additional pollen from replicates of the same treatment with the same sowing date to reach the minimum (20 mg) amount of pollen required for stoichiometric analyses.

Pollen samples were stored at −4°C. They were accurately weighed and digested with nitric acid (67%, 0.2 mL) at 80°C for 1 hour, followed by a further hour with the addition of hydrogen peroxide (30%, 0.1 mL). Distilled and deionized (Type 1) water (18 mΩ, 2 mL) was added after cooling so that the resulting solution contained 2% HNO_3_ for instrument analysis. Samples were analyzed by an inductively coupled plasma mass spectrometer-QQQ (Agilent) controlled by MassHunter Workstation (version 4.5) to measure chemical elemental concentrations (Grainger et al., 2020).

### Statistical analyses

To investigate whether and how the nutritional quality of pollen changes with increasing Na concentration in pollen, we first analyzed whether pollen samples obtained from different cultivars and various Na treatments differed in their stoichiometric phenotypes [proportions of elements; also called elementome (Peñuelas et al., 2019; Fernández-Martínez, 2022)] by performing permutational analysis of variance (PERMANOVA) in PAST 4.07b (Hammer et al., 2001). Furthermore, to explore how the Na concentration in pollen is related to the concentrations of the other measured elements, we performed redundancy analysis (RDA) in Canoco 5 (Smilauer and Lepš, 2014). In the case of RDA, we also performed independent analyses of variance (ANOVAs) for the 1st- and 2nd-axis scores of the RDA using Statistica 13 (TIBCO Software Inc.). The RDA showed that pollen Na concentration and pollen K concentration were highly correlated; thus, we further investigated the strength of this relationship by calculating the R^2^ value of the correlation.

## Results

**Figure 1:**
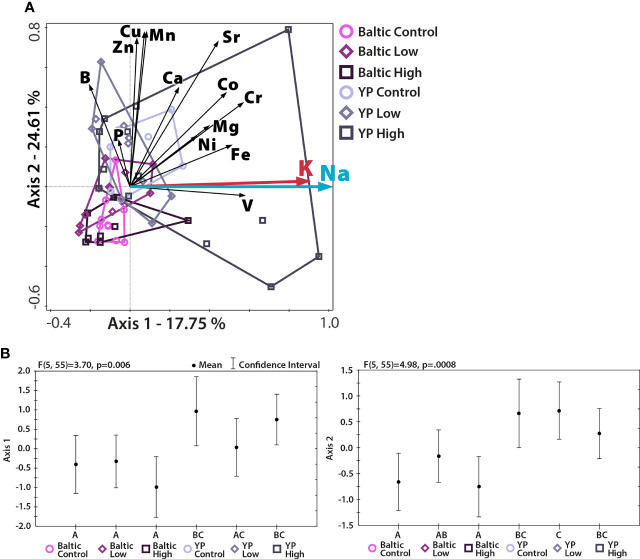
Multivariate analysis (RDA) of the relationships between the studied element concentrations and the proportion of Na in the studied pollen. N = 61, p = 0.002; **(A)** RDA plot, with the first two axes presented. The percentage of variation explained is given for both axes. The lengths of the vectors represent the contributions of the elements to the pattern shown (longer vector = greater contribution). The direction of the vector shows the axis to which it contributes. **(B)** ANOVA performed for both axis scores; different letters denote significant differences between the studied pollen groups. Different cultivars, but not Na treatments, were separated from each other. The addition of NaCl to soil increased the variation in concentrations of measured elements in pollen.

A higher concentration of Na in pollen did not translate into a higher nutritional value of pollen. Although the concentrations of the majority of studied elements were not associated with Na concentration (**Table 1** and **Figure 1**), the concentration of K was highly correlated with that of Na (**Figure 1**), meaning that an increase in the Na concentration of pollen did not correspond to a considerable change in the *K:Na* of pollen.

**Table 1:** The results of two-way PERMANOVA.

	df	F	p
**Cultivar (A)**	1	12.587	0.0001
**Na-treatment (B)**	2	1.445	0.2266
**A x B interaction**	2	0.244	0.8888
**Residual**	55		

In sunflower pollen, the proportion of 15 elements differed between cultivars and did not differ between Na treatments. There was no cultivar x Na-treatment interaction.

**Figure 2:**
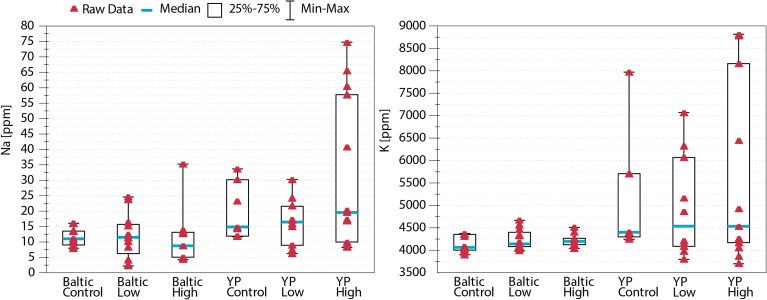
Concentrations of Na and K in the studied pollen—variance and descriptive statistics. The addition of NaCl to the soil increased the variation in both Na and K in pollen. N = 61.

**Figure 3:**
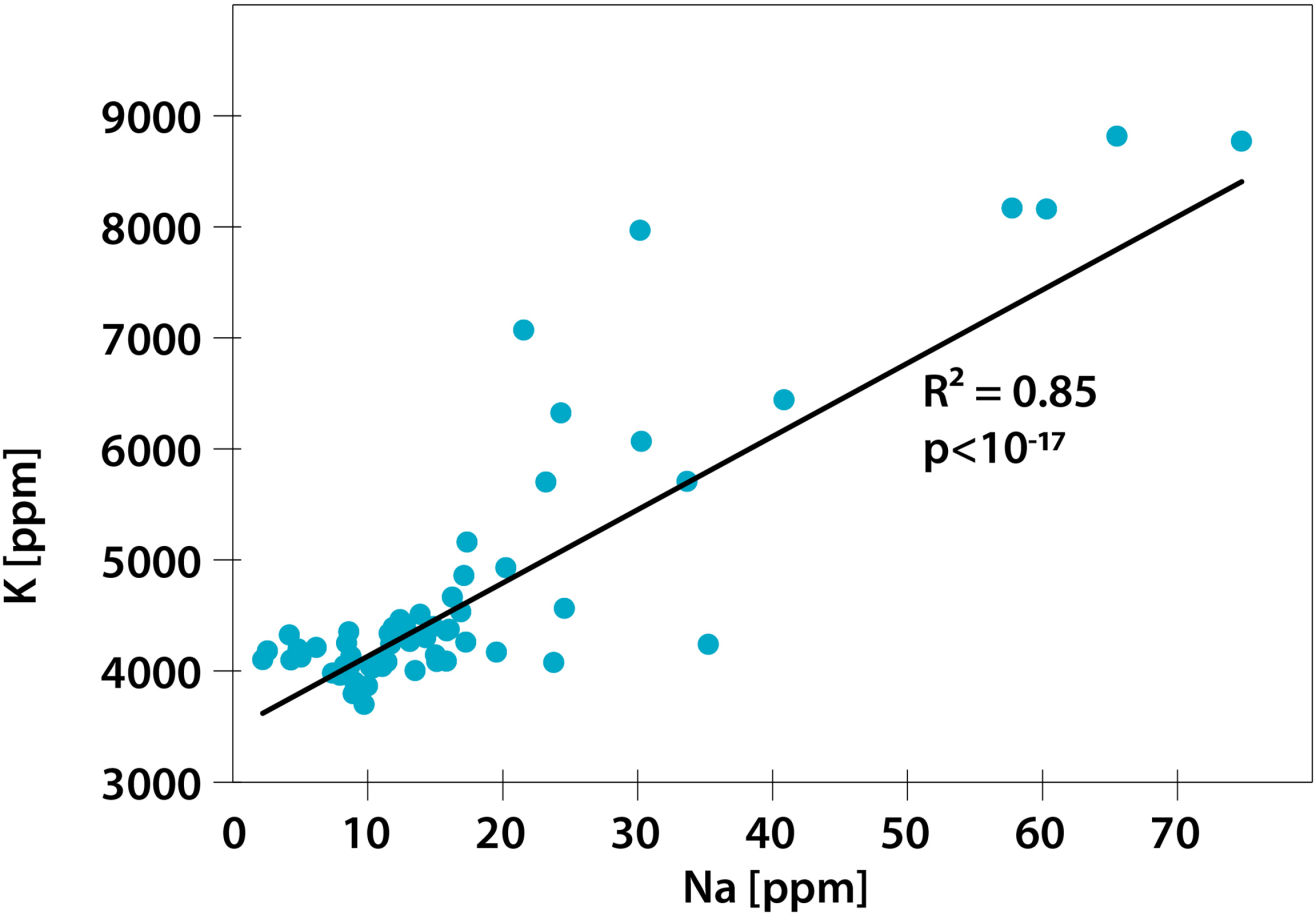
Correlation between Na and K concentrations in the studied pollen. N=61.

Regarding multielemental composition, the two sunflower cultivars had significantly different (p<0.05) levels of nutritional quality, as reflected by the proportions of the studied nutritional elements (**Table 1** and **Figure 1**), but within cultivars, the multielemental composition did not differ among the Na treatments (**Figure 1**). The majority of the observed variance (24.61%) was associated with the 2^nd^ RDA axis and was driven by manganese (Mn, 2^nd^ axis loading: 0.78), copper (Cu, 0.77), zinc (Zn, 0.74), and strontium (Sr, 0.73). The 1^st^ axis, associated with Na concentration (independent variable in the RDA), explained 17.75% of the observed variance. Additionally, the K concentration also strongly contributed to this pattern (1^st^ axis loading: 0.87). Regarding the Na and K concentrations, the YP cultivar was richer in these two elements than the Baltic cultivar, and the addition of Na (NaCl treatments: low and high) increased the variation in the concentrations of these two elements in the pollen of both cultivars (**Figures 1**, **2**). **Figure 2** shows the Na and K concentrations and descriptive statistics (median, interquartile range, min-max values) for these two elements in collected samples of pollen. **Figure 3** shows the correlation between Na and K concentrations. The average *K:Na* ratio calculated for the concentrations was 269, and the molar average *K:Na* ratio was 158.

## Discussion

Surprisingly, we did not find evidence supporting our hypothesis that the *K:Na* ratio in sunflower pollen decreases with increasing Na concentration in pollen. The sodium concentration was correlated with the K concentration, and the pollen *K:Na* ratio remained high regardless of the Na concentration. The question arises ‘what *K:Na* ratio is optimal for pollinivorous pollinators, and how much it differs from the *K:Na* ratio measured in this study?’ Scarce literature data allow for comparison of the sunflower pollen *K:Na* with nutritional needs of two pollinivore species based on their stoichiometric phenotypes (Filipiak et al., 2017; Filipiak, 2019). The optimal food *K:Na* concentration ratio is approximately 15-20 for the Western honey bee *A. mellifera* and approximately 50-90 for the solitary wild bee *Osmia bicornis* L. (Filipiak et al., 2017; Filipiak, 2019). The *K:Na* concentration ratio in the pollen measured in this study was 15 times (*A. mellifera*) and 4 times (*O. bicornis*) higher than the optimal ratio. Therefore, we hypothesize that bees, especially *A. mellifera*, must obtain Na from sources other than pollen to decrease the overall *K:Na* ratio of their food and, in the case of *A. mellifera*, to produce nutritionally balanced jelly for their larvae. Jelly is rich in Na (Wang et al., 2016), and this high concentration of Na in jelly cannot be obtained from pollen alone (Filipiak et al., 2017).

Our findings have implications for plant-pollinator interactions that are shaped by mismatches between the nutritional demand of pollinators and the nutritional supply offered by plants (Filipiak and Filipiak, 2022; Parreño et al., 2022), resulting from different or even conflicting interests of plants and pollinators (van der Kooi et al., 2021). The feeding strategy of pollinivores contrasts with that of other herbivores due to the exceptional nutritional richness of pollen. The nutritional quality of pollen is more similar to the quality of animal tissues than other plant products, making it an excellent food (Willmer, 2011). However, if a high and stable *K:Na* ratio is a general trait of pollen, regardless of its Na concentrations, pollen is nutritionally limiting for bees, which in turn might have negative consequences for bees (Kaspari, 2020). Plants selectively absorb certain nutrients in specific proportions according to the physiological requirements of the plant, and this process is part of their environmental adaptation (Huang and Salt, 2016; Vaudo et al., 2022). Considering the minor role of Na in plant metabolism, one could expect that plants using pollen as a nutritional reward for pollinators would fill pollen with a large proportion of unnecessary Na to stimulate pollination services. This should result in decreased pollen *K:Na*. However, we did not observe the effect of low *K:Na* in sunflower pollen under various scenarios of Na fertilization of the two sunflower cultivars. One possible explanation is that high pollen *K:Na* is needed to optimize plant fertilization, embryo creation, and seed development, as K homeostasis plays an important role in pollen germination and embryo and seed development (White and Karley, 2010; Dresselhaus et al., 2016; Padmanaban et al., 2017). Indeed, the review of available data on K and Na concentrations suggests that pollen in general has a high *K:Na* ratio, similar to that of other plant tissues, even considering its high variability (Filipiak et al., 2017). It is also possible that plant taxa differ in their pollen *K:Na*, depending on pollination syndromes [it was already suggested that concentrations of vital nutrients in zoophilous plants pollen may have been shaped by the nutritional needs of their pollinators (Ruedenauer et al., 2019)]; however, this possibility has yet to be investigated. Additionally, pollinivores may supplement Na from sources other than pollen to balance the overall *K:Na* in their progeny diets (Dorian and Bonoan, 2020; Khan et al., 2021), especially if direct larval food is produced by adults, as with Western honey bees (Filipiak et al., 2017). This can take place through, for example, cannibalistic behavior, drinking animal secreta, or puddling (Simpson et al., 2006; Bänziger and Bänziger, 2010; Dangles and Casas, 2012; de la Rosa, 2014; Dorian and Bonoan, 2020). It is possible that a tradeoff occurs between producing low *K:Na* pollen for pollinators and high *K:Na* pollen physiologically optimizing fertilization. The physiological mechanism of such a tradeoff and its ecological consequences constitute an interesting topic for future studies. Importantly, this study shows that, as suggested earlier (Filipiak and Filipiak, 2022; Lau et al., 2022; Parreño et al., 2022), a holistic approach to the chemical composition of pollen, complementary to the most common currently used examination of the concentration and proportion of proteins and lipids, is necessary to develop our understanding of the nutritional ecology of bees and consequently their health.

Although there are studies reporting both potassium and sodium concentrations in plant tissues [e.g. (Zhang et al., 2012; Zhao et al., 2014)], at present, the current study is the only one providing data on pollen K and Na regulation by plants. Therefore, although we do not exclude the possibility of flexible regulation of the *K:Na* ratio in pollen by plants, we are not able to perform substantive assessment of this possibility using sunflower. Interestingly, it has been shown recently on the example of *Gentiana rigescens* Franch. ex Hemsl. that concentration of K is higher in flowers than in other plant tissues [(Zhang et al., 2020); unfortunately Na was not considered in that study]. Future studies might elucidate the role of *K:Na* nutritional limitation in the ecology and evolution of bees. Currently, we do not know the role of bees and other pollinators and their interactions with plants in nutrient cycling in food webs and ecosystems (Ollerton, 2021a; Ollerton, 2021b). Given the high amount of biomass that bees move in ecosystems with the pollen they consume, we hypothesize that bees play a pivotal role in the mobilization and movement of nutrients from the soil through pollen and nectar, into food webs. Importantly, this crucial ecosystem service is shaped by nutritional interactions between bees and their host plants, including balancing *K:Na* in bee larval diet which is based on pollen.

## Conclusions

A balanced ratio of *K:Na* in food is a necessary condition for the health and fitness of herbivores. Previous studies suggested that plants manipulate their ionomes to shape their interactions with pollinators. Considering the minor role of Na in plant metabolism, one could therefore expect that plants using pollen as a nutritional reward for pollinators would fill pollen with a large proportion of unnecessary Na to stimulate pollination services. This should result in decreased pollen *K:Na*. Contrary to expectations, we did not observe the effect of low *K:Na* in sunflower pollen under various scenarios of Na fertilization of the two sunflower cultivars. We show that the concentrations of K and Na in sunflower pollen are closely linked and an increased concentration of Na in pollen correlates with an increase in the concentration of K; therefore, the *K:Na* ratio in pollen is high irrespective of its Na concentration. High pollen *K:Na* is suboptimal for pollinivores and may be harmful for bees if additional Na is not supplemented from other food sources. It is possible that high pollen *K:Na* is needed to optimize plant fertilization, embryo creation, and seed development. Consequently, there may exist a tradeoff between producing pollen with a low *K:Na* optimal for pollinator health and development and pollen with a high *K:Na* optimal for plant fertilization and development. This observation suggests not-studied to date costs and tradeoffs related to pollinivory that may shape plant-pollinivore interactions and may influence pollination ecology.

## Data availability statement

The original contributions presented in the study are included in the article/**Supplementary Material**. Further inquiries can be directed to the corresponding author.

## Author contributions

MF: Conceptualization, Methodology, Formal analysis, Validation, Writing-Original Draft, Writing – Review & Editing, Visualization, Funding acquisition. MS: Conceptualization, Methodology, Investigation, Validation, Writing – Review & Editing, Project administration. SC: Conceptualization, Methodology, Investigation, Validation, Writing – Review & Editing, Project administration. MG: Methodology, Investigation, Validation, Writing – Review & Editing. SW: Conceptualization, Methodology, Investigation, Funding acquisition, Project administration, Supervision. All authors made critical contributions to the drafts and gave permission for publication.

## Funding

This work was funded/supported by Bayer AG, Monheim, Germany; the Bio-Protection Research Centre (bioprotection.org.nz), Lincoln University, New Zealand; the Royal Society of New Zealand’s James Cook Fellowship; Jagiellonian University, Faculty of Biology (N18/DBS/000003) and the National Science Centre, Poland (grant no. 2019/33/B/NZ8/01700).

## Conflict of interest

The authors declare that the research was conducted in the absence of any commercial or financial relationships that could be construed as a potential conflict of interest.

## Publisher’s note

All claims expressed in this article are solely those of the authors and do not necessarily represent those of their affiliated organizations, or those of the publisher, the editors and the reviewers. Any product that may be evaluated in this article, or claim that may be made by its manufacturer, is not guaranteed or endorsed by the publisher.
